# Value of myocardial deformation parameters for detecting significant coronary artery disease

**DOI:** 10.34172/jcvtr.2022.30

**Published:** 2022-09-07

**Authors:** Egle Rumbinaite, Arnas Karuzas, Dovydas Verikas, Ieva Jonauskiene, Olivija Gustiene, Arslan Mamedov, Loreta Jankauskiene, Rimantas Benetis, Remigijus Zaliunas, Jolanta Justina Vaskelyte

**Affiliations:** ^1^Department of Cardiology, Medical Academy, Lithuanian University of Health Sciences, Kaunas, Lithuania; ^2^Department of Cardiac, Thoracic and Vascular Surgery, Medical Academy, Lithuanian University of Health Sciences, Kaunas, Lithuania

**Keywords:** Dobutamine Stress Echocardiography, Coronary Artery Disease, Speckle-Tracking Echocardiography, Myocardial Deformation Imaging, Myocardial Perfusion Imaging

## Abstract

***Introduction: *** The study aimed to evaluate the diagnostic value of global and regional myocardial deformation parameters derived from two-dimensional speckle-tracking echocardiography to detect functionally significant coronary artery stenosis.

***Methods:*** Dobutamine stress echocardiography and cardiac magnetic resonance myocardial perfusion imaging (CMR-MPI) were performed on 145 patients with a moderate and high probability of coronary artery disease (CAD) and LVEF≥55%. Significant CAD was defined as>50% stenosis of the left main stem,>70% stenosis in a major coronary vessel, or in the presence of intermediate stenosis (50-69%) validated as hemodynamically significant by CMRMPI. Patients were divided in two groups: non-pathological (48.3%) vs pathological (51.7%), according to CAG and CMR-MPI results. Afterwards, off-line speckle-tracking analysis was performed to analyse myocardial deformation parameters.

***Results:*** There were no differences in myocardial deformation parameters at rest between groups, except global longitudinal strain (GLS) and global radial strain (GRS) were significantly lower in the CAD (+) group: -21.3±2.2 vs.-16.3±2.3 (*P*<0.001) and 39.7±23.2 vs. 24.5±15.8 (*P*<0.001). GLS and regional longitudinal strain rate (SR) had the highest diagnostic value at high dobutamine dose with AUC of 0.902 and 0.878, respectively. At early recovery, GLS was also found to be the best myocardial deformation parameter with a sensitivity of 78%, specificity 67%, AUC 0.824.

***Conclusion:*** Global and regional myocardial deformation parameters are highly sensitive and specific in detecting functionally significant CAD. The combination of deformation parameters and WMA provides an incremental diagnostic value for patients with a moderate and high probability of CAD, especially the combination with regional longitudinal SR.

## Introduction

 Early identification of stable CAD and its correction by medical treatment or revascularization reduces the incidence of cardiac events and improves the prognosis, but the optimal diagnostic and therapeutic strategy of these patients remains controversial.^1,2^

 The evaluation of functional relevance of coronary artery stenoses with non-invasive tests as a gatekeeper to invasive coronary angiography (CAG) remains mandatory.^3^ During the past decade, stress myocardial perfusion imaging by cardiac magnetic resonance (CMR) has emerged as an accurate technique for patients with suspected CAD, thanks to high spatial and temporal resolution and the absence of ionizing radiation.^4^

 Stress echocardiography (SE) is a well-validated and cost-effective imaging modality in the detection of CAD, and it is widely used for selecting patients for invasive CAG. Although exercise echocardiography is preferred in many cases, dobutamine stress echocardiography (DSE) offers an alternative by minimising factors that decrease image quality. However, knowing the potential limitations only moderate reproducibility has been observed with DSE even by experts.^5^ This confirms the need for a new non-invasive diagnostic test that could be highly sensitive and specific in detecting functionally significant CAD.

 2D speckle-tracking echocardiography (STE) derived deformation is a newer approach to assess myocardial deformation and may improve the diagnostic value of SE by permitting quantification of global and regional myocardial function.^6^ A clinical study by Uusitalo et al. clearly illustrated the value of 2D global longitudinal strain (GLS) separately or in combination with DSE, where STE can increase the sensitivity of SE and provide information on the location, extent, and severity of myocardial ischemia in combination with visual wall motion during DSE.^7^ Small recent studies suggest that regional longitudinal deformation parameters could also have potential in detecting CAD, while the diagnostic value of regional circumferential and radial myocardial deformation parameters to predict functionally significant CAD still are not clear.^8,9^ In the present study, we sought to describe both: global and regional longitudinal, circumferential, and radial deformation parameters, which were derived from 2D STE and wall motion score as well as to evaluate the diagnostic value of these parameters for the detection of functionally significant CAD.

## Materials and Methods

###  Study population and design

 We prospectively enrolled 170 patients who were referred to the Department of Cardiology of Lithuanian University of Health Sciences from 2013 through 2016 for investigation of stable chest pain and had moderate or high pre-test probability of obstructive CAD on the types of symptoms, age, and sex.^10^ Patients were not included in the study with any of the following criteria: acute coronary syndromes, LVEF < 55% at rest on echocardiography, wall motion abnormalities (WMA) at rest or previous myocardial infarction, valvular heart disease, previous cardiac surgery, implanted pacemaker, non-sinus rhythm, known hypersensitivity to contrast agents, mental diseases, pregnancy or breast-feeding, severe renal impairment (estimated glomerular filtration rate ≤30 ml/min/1.73m^2^), contraindications to CMR-MPI. Any other structural heart disease that could cause perfusion defects (e.g. hypertrophic cardiomyopathy) was excluded from the study by performing standard echocardiography and CMR-MPI for all patients.

 The study protocol included DSE, CMR-MPI and invasive CAG. CMR-MPI and invasive CAG were performed on average 4 ± 1 weeks after DSE. Of the 170 patients, 13 were excluded due to a poor acoustic window, 9 - due to inconclusive DSE results. Late gadolinium enhancement was found for 3 patients who were excluded from the research and 145 patients with a moderate or high probability of CAD were included in the study.

###  Image acquisition and analysis

 All patients were imaged in the left lateral decubitus position using a commercially available system (Vivid 7, GE Healthcare, Horten, Norway) with a 1.5 – 4.6 MHz transducer. Standard 2D images and Doppler data were acquired from parasternal long and short axis views and apical (four-, three- and two chambers) views, which were acquired at rest, at a dobutamine dose of 20 mg/kg/min, at peak stress, and at early recovery 1 min after stress. Echocardiographic views were saved in cine-loop format from four consecutive beats. Standard echocardiographic measurements were performed according to current recommendations.^11^ LV end-systolic volume (LVESV) and LV end-diastolic volume (LVEDV) were measured, and LVEF was calculated using Simpson’s biplane technique. Qualitative assessment of regional wall motion was performed for each patient by dividing the LV into 16 segments. Each segment was then scored individually based on its motion and systolic thickening (1 = normokinesis, 2 = hypokinesis, 3 = akinesis, 4 = dyskinesis), and wall motion score index (WMSI) was calculated as the sum of the segment scores divided by the number of segments scored. Images were analysed by at least three experienced echocardiographers blinded to the results of other investigations. The worsening of contractility induced by dobutamine in two or more adjacent segments was considered a positive result.

###  Dobutamine stress echocardiography 

 DSE was performed using a standard protocol.^12^ Beta-blockers were discontinued 48 hours, nitrates - 12 hours before the study. Intravenous dobutamine was infused through a peripheral infusion line with a mechanical pump, starting with 5 μg/kg/min and increasing to 10, 20, 30, and 40 μg/kg/min at 3-minutes intervals. If no endpoint is reached, atropine (in doses 0.25 mg up to a maximum of 2 mg) was added to the 40µg/kg/min dobutamine infusion. Electrocardiogram (ECG) and arterial blood pressure were monitored continuously. The dobutamine infusion was terminated if 85% of the maximal predicted heart rate was achieved, obvious echocardiographic positivity, severe chest pain or obvious electrocardiographic positivity. The stress test was terminated prematurely in the presence of any non-tolerable symptom or side effect.^12^

###  STE analysis

 Off-line speckle-tracking analysis (EchoPac, GE Healthcare, Horten, Norway) was performed using images obtained during DSE. Cardiac cycles associated with atrial or ventricular extrasystolic beats were excluded. To achieve optimal imaging quality for subsequent analyses, the frame rate ranged from 60 to 100 frames per second. End-systole was identified as corresponding to the aortic valve closure measured by pulsed-Doppler. For strain and strain rate (SR) measurements, the endocardial borders were traced automatically with aid of manually marked reference points at the end-systolic frame in the three apical views and parasternal short-axis LV view at the level of the papillary muscles. The quality of tissue tracking was verified by the software’s own quality control function, as well as the visual assessment of the reader. In case of poor-tracking quality, the reference points were manually readjusted until satisfactory tracking was achieved. Patients with two or more segments with poor tracking even after manual correction were excluded from further analysis. After satisfactory tracking was obtained, numerical and graphical displays of myocardial deformation parameters were generated automatically for all six segments from each view.

 Global and regional strain and SR analysis were performed. Strain and SR were measured in each of the 16 LV segments. Average strain values were calculated for the whole LV (global) and the myocardial regions subtended by major coronary arteries (left anterior descending, left circumflex, or right coronary artery). Stenosis in the left main coronary artery was considered to affect both the left anterior descending and left circumflex coronary artery regions. LV GLS was calculated from loops acquired from the apical 2-, 3-, and 4-chamber views. LV global radial strain (GRS) and LV global circumferential loops are acquired from the LV short-axis view at the level of papillary muscles. End systolic and peak systolic radial and circumferential strain and SR were measured from mid-short-axis view at rest and peak dobutamine dose. End systolic and peak systolic longitudinal strain and SR were measured from apical 4- and 2-chamber and 3-chamber views according to existing guidelines.^13,14^

###  Cardiac magnetic resonance

 CMR images were acquired using a 1.5 T MRI scanner (Magnetom Aera, Siemens AG Healthcare, Erlangen, Germany) with a dedicated 18-channel phased-array receiver coil in the supine position. Cine images were obtained with a balanced steady-state free precession (bSSFP) sequence in three long-axis views, followed by a contiguous stack of short-axis views covering the entire LV from base to apex. The following imaging parameters were used: repetition time (TR) = 5.1 ms, echo time (TE) = 1.3 ms, flip angle = 80°, in-plane spatial resolution 0.9 × 0.9 mm with a slice thickness of 8 mm and 25 phases per cardiac cycle. First-pass stress perfusion imaging was performed using a saturation-recovery prepared bSSFP sequence (TR = 2.5 ms, TE = 1.3 ms, flip angle = 12° and voxel size 2.4 × 2.4 × 8.0 mm) over 50 consecutive heart beats. The images were acquired after 4 min of 140 µg/kg/min adenosine infusion and high-rate injection of 0.1 mmol/kg gadobutrol (Gadovist®, Bayer Schering Pharma AG, Berlin, Germany). In cases of the inadequate hemodynamic response, the adenosine dose was increased up to 210 µg/kg/min.^15^ Rest first-pass perfusion images were acquired > 15 min after stress perfusion imaging. The late gadolinium enhancement (LGE) images were obtained 10 min after gadolinium injection in long- and short-axis planes. Cine and LGE images were acquired at the identical long- and short-axis orientation.

 All images were analysed using dedicated software (Syngo.via, Siemens AG Healthcare, Erlangen, Germany) following a recent consensus document for quantification of LV function and mass using CMR.^16^ LV volumes (LVEDV and LVESV) were quantified using manual planimetry of the endocardial and epicardial surface from a short-axis stack and LVEF and myocardial mass were calculated. Perfusion defects were defined as subendocardial or transmural visually dark myocardial areas when compared with remote myocardium, persisting for at least 10 frames. The stress and rest perfusion scans were reviewed simultaneously, and areas of hypoperfusion were assigned to the ventricular segments, using the standard American Heart Association (AHA) 16-segment model.^17^ Myocardial tissue was considered infarcted if the signal intensity on LGE images was > 5 standard deviations above that of the remote myocardium.

###  Invasive CAG 

 After DSE and CMR-MPI all patients underwent invasive CAG. Angiography was performed using a standard protocol with a minimum of two projections obtained per vessel distribution by two experienced cardiologists. Angiographic data were analysed by three experienced investigators blinded to the DSE and CMR-MPI results.

 Significant CAD was defined by invasive CAG as > 50% stenosis of the left main stem, > 70% stenosis in a major coronary vessel, or in the presence of intermediate stenosis (50-69%) validated as hemodynamically significant by CMR-MPI, when perfusion defect was found. The other patients that had no significant coronary artery stenosis were included in the non-pathological group (the control group, CAD (-).

###  Statistical methods and analysis 

 Statistical analysis was performed using the IBM SPSS 25.0 software package. Qualitative variables were described with their frequency ​​and relative frequency rate (%). Qualitative variables’ homogeneous distribution was evaluated by chi-square (χ2) test (χ2 or Fisher’s exact test – in case of small, expected values). For comparison of quantitative variables, the Mann Whitney U, Student’s T-test, paired Student’s T-test, and non-parametric Kruskal-Wallis test were used. The quantitative trait, which did not satisfy the conditions of normality, was described by the median (min-max).

 The strain and SR parameters as CAD indicators were evaluated by using ROC curves. A subject was assessed as positive or negative according to whether the parameter value was greater, less, or equal to a given cut-off value. Associated with any cut-off value was the probability of a true positive (sensitivity) and a true negative (specificity). A commonly used index of accuracy is the area under the ROC curve (AUC), with values close to 1.0 indicating high diagnostic accuracy. A p-value less than 0.05 was considered statistically significant.

 To describe the intra-observer variability, the same investigator re-evaluated regional longitudinal strain and SR on two separate occasions of 20 randomly selected patients at rest, as well as at high dobutamine dose. To evaluate inter-observer variability, two investigators blinded to previously obtained data, performed the same measurements. Intra-observer and inter-observer variability values were calculated as the absolute difference between the corresponding two measurements as a percent of the mean.

## Results

###  Study population

 The study population consisted of 145 individuals (68 [46.9%] men) without a history of CAD. Based on Diamond-Forrester risk score, 57.9% of the study group had moderate (15-85%) and 42.1% had high obstructive CAD risk (> 85%). LVEF was normal in all patients (mean, 58.2 ± 6.0% at rest and 62.4 ± 6.9% at peak stress). Detailed clinical characteristics are shown in Table 1. Conventional echocardiographic parameters did not differ between groups and all parameters were within normal range (Table 2). Conventional WMA was possible in 94% of the segments at rest and 90% at peak stress. STE strain measurement was feasible in 87 and 88% of the analysed 720 segments, respectively (*P* = 0.89). At peak stress, 87 vs. 75% of segments.

**Table 1:** Clinical characteristics of study population

**Characteristics**	**CAD (-) group (n=70)**	**CAD (+) group (n=75)**	***p*** ** value**
Male, n (%)	30 (42.9)	38 (50.7)	0.22
Age (years)	62.6 ± 8.8	64.9 ± 7.6	0.34
History of hypertension, n (%)	32 (45.7)	31 (41.3)	0.16
History of smoking, n (%)	23 (32.8)	22 (29.3)	0.72
Obesity, n (%)	35 (50.0)	27 (36.0)	0.17
Diabetes, n (%)	11 (15.7)	8 (10.7)	0.24
Family history of CAD, n (%)	30 (42.9)	17 (22.7)	0.23
*Moderate CAD probability, n (%)	49 (70.0)	35 (46.7)	0.21
*High CAD probability, n (%)	27 (38.5)	34 (45.3)	0.44

*CAD probability assessment by Diamond-Forrester classification (1979).

**Table 2:** 2D and tissue Doppler echocardiography parameters

**Characteristics**	**CAD (-) group**	**CAD (+) group**	***p*** ** value**
LVEDD (mm)	45.4 ± 5.8	45.6 ± 6.7	0.72
LVEDD index (mL/m^2^)	23.1 ± 2.5	23.6 ± 3.7	0.77
LVESD (mm)	32.3 ± 6.5	30.5 ± 6.6	0.38
LVESD index (mL/m^2^)	16.2 ± 3.3	15.4 ± 3.6	0.83
LVEDV (mL)	94.1 ± 25.4	89.3 ± 25.5	0.35
LVEDV index (mm/mL^2^)	45.7 ± 10.4	44.8 ± 13.3	0.90
LVESV (mL)	39.1 ± 14.2	36.8 ± 15.1	0.76
LVESV index (mL/m^2^)	19.2 ± 6.4	18.1 ± 7.6	0.34
LVEF (%)	59.2 ± 6.3	60.1 ± 6.8	0.81
MMI (g/m^2^)	84.8 ± 20.6	79.9 ± 20.1	0.44
RWT	0.36 ± 0.12	0.38 ± 0.11	0.74
E/A ratio*	0.9 ± 0.3	0.9 ± 0.2	0.75
E peak velocity (cm/sec)	60.7 ± 14.3	64.5 ± 18.1	0.32
A peak velocity (cm/sec)	70.2 ± 25.2	74.4 ± 12.2	0.19
e’ lateral (m/sec)	9.2 ± 1.6	9.2 ± 2.2	0.59
e' septal (m/sec)	7.5 ± 1.8	8.9 ± 5.3	0.74
E/e’	7.3 ± 1.2	7.1 ± 2.4	0.63
DT (ms)	275.4 ± 56.6	270.3 ± 75.3	0.26
LA max (mL/m^2^)	28.2 ± 4.3	29.3 ± 3.6	0.47
WMSI at rest	1.00	1.00	1.00
WMSI at peak dose	1.02 ± 0.1	1.16 ± 0.5	0.12
WMSI at recovery phase	1.02 ± 0.2	1.09 ± 0.4	0.26

Abbreviations: LVEDD, left ventricular end-diastolic diameter; LVESD, left ventricular end-systolic diameter; LVEDV, left ventricular end-diastolic volume; LVESV, left ventricular end-systolic volume; LVEF, left ventricular ejection fraction; MMI, myocardial mass index; RWT, relative wall thickness; LA, left atrium; LA, left atrium volume; LA vol. index, left atrial volume index; E/A, early diastolic transmitral flow velocity (E) and atrial systolic velocity (A) ratio; E, early diastolic transmitral flow velocity; A, atrial systolic velocity; e’, early diastolic mitral annular velocity; E/e’, early diastolic transmitral flow velocity and early diastolic mitral annular velocity; LA, left atrium; WMSI,wall motion score index; DT, deceleration time.

 Invasive CAG was performed on all 145 patients. CAG detected stenosis ≥70% in 75 patients. There were also 9 cases in which CAG confirmed ≥50% stenosis of the left main stem. Other angiographic findings of patients are shown in Table 3.

**Table 3:** Characteristics of CAG findings*

**Characteristics**	**CAD (+) group** **(n=75)**
Single vessel disease n (%)	35 (46.7%)
Two vessels disease n (%)	19 (25.3%)
Three vessels disease n (%)	21 (28.0%)
Diseased vessel left main coronary artery n (%)	9 (12.0%)
Diseased vessel right coronary artery n (%)	31 (41.3%)
Diseased vessel left circumflex artery n (%)	18 (24.0%)
Diseased vessel left anterior descending artery n (%)	44 (58.7%)

*Significant coronary artery stenosis was defined as 70% or greater luminal narrowing of epicardial coronary vessels.

###  CMR-MPI findings

 All patients were evaluated by conventional cardiac magnetic resonance (CMR) and adenosine stress magnetic resonance. Conventional CMR measurements are shown in Table 4. There were no significant differences in conventional parameters between the groups at rest. After CMR-MPI perfusion, defects were found in 71 (48.9%), no defects in 74 (51.1%) patients. Although four patients had > 70% stenosis on CAG (agreement - kappa (ź) = 0,87), they had no perfusion defects. There were 48 (67.6%) defects in the left anterior descending coronary artery territory, 33 (46.5%) in the left circumflex artery territory, and 35 (49.3%) in the right coronary artery territory. In vessel-based analysis, CMR-MPI had a sensitivity of 93%, specificity of 87% and global accuracy of 0.947.

**Table 4:** Conventional CMR parameters

**Characteristics**	**CAD (-) group**	**CAD (+) group**	***P*** ** value**
LVEDD (mm)	43.7 ± 5.4	45.4 ± 5.6	0.68
LVESD (mm)	34.3 ± 6.5	32.5 ± 6.7	0.47
LVdiastolic volume index (mL/m^2^)	74.5 ± 18.4	80.8 ± 20.3	0.64
LV systolic volume (mL)	96.4 ± 21.2	102.2 ± 23.4	0.75
LV systolic volume index (mL/m^2^)	31.4 ± 6.4	32.3 ± 7.7	0.54
LVEF (%)	59.3 ± 6.1	60.3 ± 6.8	0.91
MMI (g/m^2^)	82.8 ± 20.4	79.8 ± 20.9	0.36
LW (mm)	9.3 ± 2.2	9.5 ± 2.9	0.64
IVP (mm)	10.0 ± 3.3	10.3 ± 3.2	0.85
LA area (cm^2^)	24.2 ± 4.4	23.3 ± 4.6	0.67

Abbreviations: LW, lateral wall; IVP, interventricular septum; other abbreviations explained in previous tables.

###  DSE findings according to both conventional and novel deformation parameters

 There were 60 positive and 85 negative DSE results. The sensitivity and specificity of inducible WMA for detecting myocardial ischemia were 78% and 83% (AUC 0.767) at peak dose. Regional deformation parameters were compared according to vessel-based analysis.

###  Regional and global myocardial strain, systolic, early, and late diastolic SR at rest

 There were no differences in peak systolic, early, and late diastolic longitudinal, circumferential, radial strain and SR parameters at rest between the groups (Table 5) except GLS and GRS were significantly lower in CAD (+) group at rest.

###  Regional and global myocardial strain, systolic, early, and late diastolic SR at high dobutamine dose

 Regional longitudinal and radial strain at high dobutamine dose were significantly lower for patients in the CAD (+) group (Table 5). GLS and GRS in CAD (+) group remained significantly lower during high doses as at rest, and global circumferential strain (GCS) became significantly lower in the CAD (+) group. There were no significant differences in regional circumferential strain at high dose between groups (Table 5).

**Table 5:** Global and regional myocardial strain and SR parameters

**Strain, SR parameters**	**CAD (-) group**	**CAD (+) group**	***P*** ** value**	**CAD (-) group**	**CAD (+) group**	***p*** ** value**	**CAD (-) group**	**CAD (+) group**	***P*** ** value**
	**Rest**			**High Dobutamine Dose**			**Early Recovery Phase**		
Regional myocardial strain, %									
Longitudinal strain	-19.6 ± 2.3	-18.7 ± 3.2	0.38	**-**20.2 ± 3.8	-16.1 ± 3.6	**<0.001**	**-**19.6 ± 3.7	-16.5 ± 3.1	**0.01**
Circumferential strain	-18.6 ± 5.8	-19.5 ± 8.1	0.52	-21.8 ± 5.6	-19.9 ± 8.6	0.21	-21.6 ± 5.3	-19.2 ± 8.1	0.16
Radial strain	41.7 ± 28.2	29.9 ± 18.3	0.16	28.5 ± 14.5	16.9 ± 15.9	**<0.001**	26.5 ± 14.5	16.5 ± 15.9	**<0.001**
Strain rate (SR), s^-1^									
Longitudinal SR	-1.3 ± 0.3	-1.3 ± 0.2	0.51	-2.3 ± 0.3	-2.0 ± 0.4	**0.03**	-2.3 ± 0.2	-2.0 ± 0.4	**0.02**
Circumferential SR	-1.8 ± 0.5	-2.0 ± 0.6	0.42	-3.3 ± 0.9	-2.7 ± 1.2	**0.04**	-3.2 ± 0.8	-2.5 ± 1.1	**0.02**
Radial SR	2.3 ± 0.6	2.2 ± 0.8	0.55	4.2 ± 1.6	3.5 ± 1.3	**0.01**	4.0 ± 1.8	3.6 ± 1.4	**0.04**
Early diastolic strain rate (SR), s^-1^									
Longitudinal SR	1.4 ± 0.3	1.2 ± 0.3	0.54	1.7 ± 0.3	1.4 ± 0.2	**0.04**	1.7 ± 0.4	1.5 ± 0.3	**0.04**
Circumferential SR	1.8 ± 0.7	1.6 ± 0.8	0.34	2.7 ± 0.8	2.4 ± 0.9	0.24	2.5 ± 0.8	2.5 ± 0.6	0.56
Radial SR	-2.0 ± 1.3	-1.8 ± 1.2	0.63	-2.6 ± 1.7	-2.1 ± 2.2	**0.03**	-2.6 ± 1.5	-2.2 ± 2.0	**0.04**
Late diastolic strain rate (SR), s^-1^									
Longitudinal SR	1.4 ± 0.2	1.3 ± 0.2	0.32	1.9 ± 0.3	1.5 ± 0.5	**0.04**	1.7 ± 0.4	1.6 ± 0.5	0.33
Circumferential SR	1.3 ± 0.5	1.3 ± 0.4	0.54	2.4 ± 0.7	2.1 ± 0.6	0.44	2.2 ± 0.8	2.1 ± 0.5	0.44
Radial SR	-1.9 ± 0.6	-2.0 ± 0.8	0.86	-2.5 ± 1.6	-2.2 ± 1.7	0.86	-2.5 ± 1.1	-2.3 ± 1.8	0.75
Global myocardial strain, %									
Global longitudinal strain	-21.3 ± 2.2	-16.3 ± 2.3	**<0.001**	-24.1 ± 2.6	-17.2 ± 2.2	**<0.001**	-23.5 ± 2.5	-17.5 ± 2.2	**0.003**
Global circumferential strain	-19.7 ± 5.4	-18.4 ± 7.1	0.11	-21.7 ± 5.1	-17.5 ± 6.2	**0.03**	-21.1 ± 3.8	-17.2 ± 5.3	**0.03**
Global radial strain	39.7 ± 23.2	24.5 ± 15.8	**<0.001**	41.5 ± 24.1	25.3 ± 16.4	**<0.001**	40.3 ± 18.7	24.8 ± 17.5	**0.002**

 All projections systolic SR parameters at high dobutamine dose were significantly lower for patients in the CAD (+) group (Table 5).

 Regional longitudinal and radial early diastolic SR parameters significantly decreased in CAD (+) group patients and were significantly lower. Circumferential early diastolic SR showed no statistically significant change from rest to high dose in both groups and had no significant difference between the groups. Only longitudinal late diastolic SR showed statistically significant change from rest to high dose and was significantly lower in CAD (+) group patients. Circumferential and radial late diastolic SR had no significant changes and did not differ between the groups (Table 5).

###  Regional and global myocardial strain, systolic, early, and late diastolic SR at recovery

 All global and regional longitudinal and radial strain parameters at early recovery phase remained lower for CAD (+) group patients (Table 5). Circumferential strain showed the same tendency as at high doses and had no significant difference between the groups.

 Regional systolic SR at early recovery phase was lower for CAD (+) group patients (Table 5). Regional longitudinal and radial early diastolic SR parameters remained decreased in CAD (+) group at early recovery phase, and circumferential early diastolic SR showed the same tendency as at high doses and had no significant difference between the groups. All late diastolic SR parameters at early recovery phase had no significant differences between the groups (Table 5).

###  ROC analysis of stress speckle-tracking parameters 

**Figure 1:**
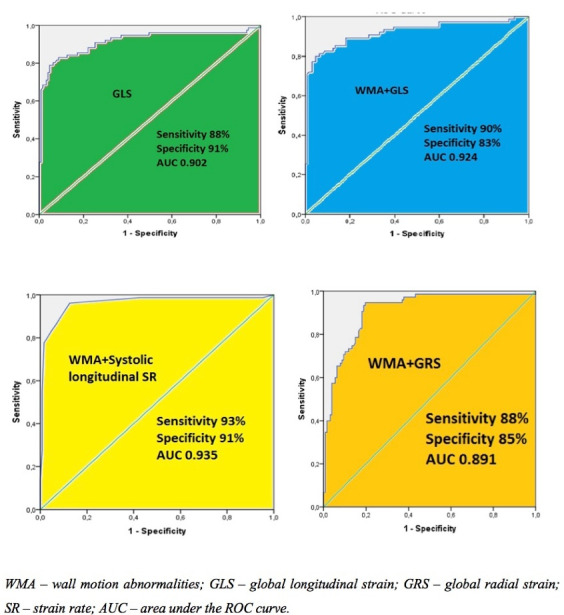
Combining speckle-tracking and visual wall motion analysis to assess functionally significant CAD.

 The greatest diagnostic accuracy for hemodynamically significant stenosis had GLS at high dobutamine doses (sensitivity 88%, specificity 91%, AUC 0.902) (Figure 1). Moreover, regional longitudinal systolic SR was a good predictor, and its diagnostic accuracy did not differ significantly from GLS (AUC – 0.878 vs. 0.902, *P* = 0.741). The third best parameter was GRS at high dobutamine doses with an AUC of 0.854 with a sensitivity of 84%, specificity of 78%. Radial systolic SR, also, showed the same tendency to have a good diagnostic value and had AUC of 0.835 (sensitivity 77%, specificity 71%). Diagnostic accuracy of global and regional systolic circumferential parameters was significantly lower compared to longitudinal and radial but had significant value for ≥ 70% CA stenosis prediction (AUC - GCS 0.704 vs. GLS 0.902, *P* = 0.03; GCS 0.704 vs. GRS 0.854, *P* = 0.045; systolic parameters AUC: circumferential 0.716 vs. longitudinal 0.878, *P* = 0.032; circumferential 0.716 vs. radial 0.835, *P* = 0.048; other results of these parameters are shown in Table 6**).** According to ROC analysis of diastolic deformation parameters, longitudinal and radial early diastolic SR parameters had sufficient predictive value for detecting CAD with longitudinal early diastolic SR having the greatest AUC of 0.778 (sensitivity 76%, specificity 64%). Radial early diastolic SR sensitivity was 72%, specificity 65%, AUC 0.735. Circumferential early diastolic SR had no significant predictive value (AUC 0.667). Radial and circumferential late diastolic SR had the same tendency and had no significant diagnostic accuracy for detecting CAD, but longitudinal late diastolic SR showed to be a significant predictor and had a similar diagnostic value as radial early diastolic SR (sensitivity 73%, specificity 61%, AUC – 0.729).

**Table 6:** ROC analysis of deformation parameters.

**Strain, SR parameters**	**Cut-off value**	**Sensitivity**	**Specificity**	**AUC**	***P*** ** value**	**Cut-off value**	**Sensitivity**	**Specificity**	**AUC**	***P*** ** value**
	**High Dobutamine Dose**					**Early Recovery Phase**				
Regional myocardial strain, %										
Longitudinal strain	-19.1	74	68	0.755	**0.02**	-18.2	70	67	0.736	**0.03**
Circumferential strain	-21.2	62	55	0.646	0.26	-19.9	61	58	0.627	0.27
Radial strain	22.4	76	59	0.721	**0.03**	21.1	68	65	0.701	**0.04**
Strain rate (SR), s^-1^										
Longitudinal SR	-2.1	83	79	0.878	**0.003**	-2.1	80	66	0.779	**0.01**
Circumferential SR	-2.9	72	65	0.716	**0.04**	-2.8	69	60	0.689	0.35
Radial SR	3.6	77	71	0.835	**0.001**	3.8	77	64	0.726	**0.03**
Early diastolic strain rate (SR), s^-1^										
Longitudinal SR	1.6	76	64	0.778	**0.02**	1.6	78	67	0.729	**0.04**
Circumferential SR	2.5	68	52	0.667	0.44	2.5	65	54	0.647	0.64
Radial SR	-2.4	72	65	0.735	**0.02**	-2.5	73	69	0.735	**0.03**
Late diastolic strain rate (SR), s^-1^										
Longitudinal SR	1.8	73	61	0.729	**0.04**	1.6	70	64	0.673	0.09
Circumferential SR	2.1	59	51	0.615	0.34	2.1	54	49	0.585	0.64
Radial SR	2.3	66	62	0.684	0.16	2.4	61	60	0.644	0.19
Global myocardial strain, %										
Global longitudinal strain	-20.1	88	91	0.902	**<0.001**	-19.7	78	67	0.824	**0.002**
Global circumferential strain	-20.4	71	63	0.704	**0.04**	-19.2	63	57	0.628	0.11
Global radial strain	33.6	84	78	0.854	**0.004**	31.4	75	72	0.767	**0.01**

 GLS as the greatest CAD predictor was found analysing images at early recovery phase, but the diagnostic value was lower compared to high dobutamine dose phase evaluation (0.824 vs. 0.902, sensitivity 78%, specificity 67%). The second-best diagnostic parameter evaluating the recovery phase was, also regional longitudinal systolic SR (sensitivity 80%, specificity 66%, AUC 0.779). GRS and radial systolic SR showed the same tendency to be good predictors as in high doses phase (GRS - 0.767; radial systolic SR – 0.726). The evaluation of regional and global circumferential parameters at early recovery phase revealed that diagnostic value significantly decreased and became not sufficient (AUC values, sensitivity and specificity showed in Table 6). The Analysis of diastolic SR parameters showed that only longitudinal early diastolic SR had sufficient diagnostic value for detecting CAD, other early and late diagnostic SR parameters had no significant predictive value.

###  Reproducibility of speckle-tracking 

 Interobserver agreements for GLS were 5% and 3% at rest, 6% and 4% at peak stress, and 8% and 6% at recovery. Intraobserver agreement for GLS was 6% and 3% at rest, 7% and 7% at peak stress, and 5% and 4% at recovery.

 Interobserver agreement for regional longitudinal strain and SR were 7% and 3% at rest, 6% and 3% at peak stress, and 8% and 6% at recovery. Intraobserver agreement for regional strain and SR were 7% and 4% at rest, 8% and 8% at peak stress, and 5% and 6% at recovery.

 Intra-observer agreement for WMA was 9 and 7%, respectively. Agreement between the two readers for the two different methods was 10 and 11%, respectively.

###  Additional diagnostic value of combining speckle-tracking and visual wall motion analysis for the assessment of functionally significant CAD

 According to ROC analysis of combination of WMA and speckle-tracking parameters during high dobutamine doses increased diagnostic value for detecting significant CAD. The analysis of separate parameters showed that the highest AUC increase was detected in the combination of longitudinal systolic SR and WMA – 0.935, sensitivity 93%, specificity 91%. Other significant combinations were with GLS and GRS which diagnostic accuracy increased up to 0.924 and 0.891, respectively (Figure 1). Other deformation parameters combined with WMA had no significant diagnostic value increase.

## Discussion

###  Major findings

 In the present study, we examined global and regional cardiac function using 2D STE and evaluated their diagnostic value to detect functionally significant coronary artery stenosis in patients with a moderate and high probability of CAD. There are two main findings in our study. Firstly, our data show that global and regional longitudinal, radial myocardial deformation parameters peak dobutamine dose and at early recovery are sensitive and specific in predicting functionally significant, associated with perfusion defects, coronary artery stenosis, while the value of circumferential myocardial deformation parameters is only moderate. Moreover, GLS of LV derived from STE was observed to be the best predictor with highest accuracy of > 70% coronary artery stenosis at high dobutamine dose. Secondly, the combination of speckle-tracking parameters and conventional visual wall motion analysis can provide additional value in detecting significant CAD.

###  The role of stress myocardial deformation parameters in assessing myocardial ischaemia

 Myocardial deformation imaging utilizing STE enables quantification of global and regional myocardial function and evaluation of LV longitudinal, circumferential, and radial motion.^18^ Recent studies identify the diagnostic power of STE even at rest. Montgomery D. et al. reported useful information about global and regional myocardial functions at rest when endocardial thickening visually was normal, where the cut-off values of global and regional strain and SR parameters predicted presence of > 50 % stenosis at rest with similar diagnostic accuracy as conventional wall motion scoring, furthermore GLS sensitivity and specificity (66/76%) for detecting CAD was comparable to WMSI measured during stress.^19^ Our study demonstrated a similar tendency and GLS and GRS were significantly decreased in the pathologic group even at rest, but regional deformation parameters had no significant difference.

 A systematic review of diagnostic value of longitudinal deformation parameters to predict coronary artery stenosis demonstrated that GLS measurements during dobutamine stress resulted in a far better diagnostic accuracy for CAD than the resting values, and the best results showed evaluation in high dobutamine dose with sensitivity 84% and specificity 88%.^20^ Compared with our study results, a higher accuracy of GLS was found and this fact further increased the clinical value of this parameter. The other study of G. Dattilo et al. modified the GLS parameter and calculated deltaGLS ([(stress strain – rest strain)/rest strain] x100).^21^ ROC analysis showed that deltaGLS had high accuracy with AUC 0.916 in distinguishing positive and negative coronary CT angiography groups and predicting CAD, but our study CAD improves the probability of CAD with more sensitive test - CMR-MPI.

 Since GLS value has been demonstrated in many studies, recent clinical trials have been focused on regional deformation parameters, where functional confirmation of the hemodynamic significance of coronary stenosis is by FFR and/or PET imaging.^7^ Our results are in line with several previous studies showing that strain and SR imaging during DSE are useful for the detection of acute and stable CAD.^22-26^ Regional longitudinal parameters showed a good potential in detecting CAD, however, the heterogeneity for obtaining regional data remains a challenge.^20^ Shimoni S et al. study demonstrated the comparison of global and regional longitudinal strain and showed that GLS has a higher accuracy for detecting significant stenosis (AUC – 0,80 vs 0,76).^27^

 The research of Li L. investigated the relationship between three main projections of LV myocardial mechanics (longitudinal, radial, circumferential) evaluated by speckle-tracking echocardiography and degree of coronary artery stenosis.^28^ They found the tendency that these strain measurements can predict > 50% and > 75% stenosis, but with a different accuracy. The highest diagnostic value for severe stenosis had the same parameters as in our study – GLS and GRS with AUC of 0.896 and 0.866, respectively. Circumferential parameters had significantly lower diagnostic accuracy compared with longitudinal and radial, especially, predicting > 50% stenosis (GCS 0.723 vs. GLS 0.899 vs. GRS 0.873). The other study showed greater longitudinal and radial value than circumferential – MY. Xie et al. analysis of patients with multivessel CAD.^29^ This study found a significant decrease only of longitudinal and radial deformation parameters in patients with multivessel CAD, but circumferential parameters had no significant difference.

 Visual WMA analysis is subjective and considerable expertise is required to achieve high diagnostic accuracy. The quantitative analysis of myocardial deformation by strain and SR imaging in combination with WMA may help to provide an incremental value for CAD detection. The same Finland scientist study demonstrated that STE parameters evaluated in different phases of dobutamine stress (at low, high doses and early recovery phase) in combination with WMA provided additional value over visual analysis alone.^7^ Isolated WMA diagnostic accuracy was 0.68, in combination with STE parameters in high dose – WMA + strain 0.70, WMA + SR 0.71, WMA + PSI 0.69, at recovery phase – WMA + strain 0.75, WMA + SR 0.78, WMA + PSI 0.79. These findings agree with our study that SR can provide the highest additional diagnostic value in combination with WMA. One of the main multicentre study which analysed the incremental value of 2D speckle-tracking strain imaging to wall motion analysis for detection of CAD, found that the combination longitudinal strain and WMA had the highest sensitivity, specificity, and accuracy (100%, 87.5%, and 0.963 respectively).^24^ The results of combinations with circumferential and radial strain parameters were: sensitivities - 73.9%, 78.3%, specificities - 78.6%, 57.1%, accuracies – 0.757, 0.703, respectively. As the study showed, the longitudinal strain analysis had a higher diagnostic accuracy than circumferential and radial strains. The differences between this and our study looking at radial projection can be determined by a smaller number of patients and using no test of validation of functionally significant coronary artery stenoses in this study.

###  The importance of functionally significant coronary artery stenoses

 The strength of our study is that all patients had functional coronary artery stenosis confirmation using CMR-MPI, while most of the previous studies did not analyse haemodynamic significance of coronary stenosis. The most accurate method for the measurement of haemodynamically significant CAD is FFR during diagnostic CAG, however, it is an expensive and invasive method.^30,31^ Dagdelen et al. demonstrated that both strain and SR correlated with FFR within the left coronary system.^32^ In another study, haemodynamic significance of intermediate coronary lesions was assessed by Weidemann et al., where SR was the best predictor with a sensitivity and specificity of 89% and 86% respectively.^26^

 In recent studies, it was revealed that CMR has excellent diagnostic performance in the detection of functionally significant CAD defined according to gold standard FFR. Kamiya K. et al. reported the highest overall sensitivity of CMR compared with PET and WMA diagnosed on DSE.^33^ Indeed, recently published meta-analysis of 3788 patients from 23 studies, the sensitivity of CMR at the patient-level were 90% (95% confidence interval, 74 to 97), specificity 94% (95% confidence interval, 79-99) and pre-vessel diagnostic performance sensitivity was 91% (95% confidence interval, 84-95).^34^ Moreover, few studies compared CMR with FFR. The first study enrolled 103 patients with intermediate or high pre-test probability of CAD showed very good accuracy of functionally significant CAD as assessed by FFR.^35^ Another study by Watkins S. et al. also proved that CMR can show excellent sensitivity, specificity, and positive and negative predictive values compared with FFR.^36^ Optimization of standardized CMR protocols now places this method as a potential first-line modality of suspected CAD, however, the adoption of this technique has been largely limited to major centres.^37^

 Our study results show that STE-derived GLS helps to assess myocardial ischaemia with very high sensitivity and specificity. Even slight changes and myocardial dysfunction caused by a single diseased vessel could be detected with STE, so it could be also a beneficial tool when there are contraindications to cardiac CMR.

 However, our study had some limitations regarding the methodology. Firstly, one of the main limitations of our study is that our protocol did not include FFR as a reference standard for the functional assessment of coronary artery stenosis. According to a recent meta-analysis, CMR-MPI had the highest performance for diagnosis of ischaemia-causing CAD when directly compared with FFR.^34^ Thus, we relied on a CMR-MPI for evaluation of the functional severity of coronary stenoses. Secondly, single-centre character and the assessment of strain and SR confined to a single vendor of echocardiographic equipment may also be considered as a limitation of our study. Moreover, contrast imaging during DSE, which could improve the diagnostic value of DSE, was not used in our study. As it was demonstrated by Nagy et al., both tissue velocity and speckle-tracking-based myocardial deformation analysis are feasible during contrast-enhanced DSE, but they failed to demonstrate a clear diagnostic benefit of additional strain analysis over expert wall motion scoring alone.^38^ Another limitation is that strain and SR can be related to some other cardiac disease than CAD, e.g. arterial hypertension. However, the prevalence of this CAD risk factor did not differ significantly between groups (p = 0.18). Moreover, all patients in our study had normal LVEF and no evidence of other structural heart diseases. Therefore, our results may not apply to patients with cardiac conditions other than CAD.

 Lastly, we excluded 13 patients with the suboptimal echocardiographic visualization assessment of the endocardial border. However, this is in line with recently published data focusing on the reproducibility of this technique in a general DSE population and should also be considered in light of the 8% of DSE studies that are uninterpretable for conventional visual analysis.^39,40^ Generally, SR measurement is more sensitive to noise, curves are more complex, and it is quite often difficult to interpret them compared to strain curves. Therefore, a skilled professional is needed to identify SR parameters, and those professionals are not available in every echo lab. Thus, this is a significant limitation in daily clinical practice.

## Conclusion

 This is one of the first studies showing that global and regional myocardial deformation parameters are highly sensitive and specific in detecting hemodynamically significant, validated by perfusion defects, coronary artery stenosis in patients with moderate and high probability of CAD. However, global deformation parameters should be selected as a higher diagnostic accuracy having indicators. Despite high myocardial strain and SR accuracy, the combination of deformation parameters and WMA can provide an incremental value for CAD detection, especially the combination with systolic SR.

## Acknowledgements

 All authors played a key role in the study.

## Funding

 This research was funded by a grant (No. MIP-037/2013) from the Research Council of Lithuania.

## Ethical approval

 This study was approved by the Kaunas Regional Biomedical Research Ethics Committee (No. BE-2-21). Written informed consent was obtained from all patients.

## Competing Interest

 The authors declared no potential conflicts of interest with respect to the research, authorship, and publication of this article.
